# Micro Electro-Osmotic Thrusters of Power-Law Fluids for Space Propulsion

**DOI:** 10.3390/mi14050949

**Published:** 2023-04-27

**Authors:** Jiaxuan Zheng, Jialu Wang, Yongjun Jian

**Affiliations:** 1 College of Mathematics Science, Inner Mongolia Normal University, Hohhot 010022, China; zhengjx@mail.imu.edu.cn; 2 Center for Applied Mathematical Science, Hohhot 010022, China; 3 School of Mathematical Science, Inner Mongolia University, Hohhot 010021, China; 32036044@mail.imu.edu.cn

**Keywords:** electro-osmotic thrusters, power-law fluids, non-Newtonian fluids, microchannel

## Abstract

In this article, electro-osmotic thrusters (EOTs), which are full of non-Newtonian power-law fluids with a flow behavior index *n* of the effective viscosity, are theoretically investigated in a microchannel. Different values of the flow behavior index represent two kinds of non-Newtonian power-law fluids, pseudoplastic fluids (*n* < 1) and dilatant fluids (*n* > 1), which have not yet been considered to be used as propellants in micro-thrusters. Analytical solutions of the electric potential and flow velocity are obtained using the Debye–Hückel linearization assumption and the approximate scheme of hyperbolic sine function. Then, thruster performances of power-law fluids, including specific impulse, thrust, thruster efficiency, and thrust-to-power ratio, are explored in detail. Results show that these performance curves strongly depend on the flow behavior index and electrokinetic width. It is noted that the non-Newtonian pseudoplastic fluid is most suitable as a propeller solvent in micro electro-osmotic thrusters owing to its improving or optimizing deficiencies in the performances of the existing Newtonian fluid thrusters.

## 1. Introduction

In the era of rapid development in aerospace engineering, micro/nano spacecrafts have widely been required on account of their inexpensive launch costs, as well as small energy dissipation and low space mission risks, to perform high-resolution measurements and complex and multifunctional assignments in the space environment [1,2,3,4,5]. The requirement has facilitated the development of micro/nano thrusters in which electro-osmosis technology is comprehensively applied, owing to enabled liquid intensive delivery, flow control, as well as mass transfer enhancement [6,7,8,9,10,11,12,13]. Electro-osmotic flow (EOF) in the thrusters is closely related to the electric double layer (EDL), which is generated by the interaction between an electrolyte solution and surface charges on the channel walls. When an external electric field is applied along channel surfaces, the mobile ions in the EDL are actuated by the electrostatic body force so that the liquid moves with them due to the viscosity of the fluid. This is the well-known electro-osmotic flow (EOF). Diez et al. [14] first introduced the electro-osmotic flow through a nanochannel in a space propulsion study, which provided a theoretical basis for the novel class of micro/nano electro-osmotic thrusters. Huang and Huang [15] further combined nano electrokinetic thrusters with two-liquid electro-osmotic flows and analyzed performances of the thrusters under the small wall zeta potential. Zheng and Jian [16,17] investigated the influence of the soft nanochannel on the electro-osmotic thrusters and found that the thruster could achieve an efficiency as high as 90% and deliver a thrust of about 0–20 μN.

In the above-mentioned research, the fluids of thrusters are deemed Newtonian fluids, while biological fluids, such as blood, saliva, DNA, and polymer solutions, have not yet been systematically considered in the study of electrokinetic thrusters, which are thought of as non-Newtonian fluids. Hence, in the present work, power-law fluids, a most popular non-Newtonian fluid, are brought in as a propellant driven by the electro-osmotic body force through a microchannel. Such new propulsive materials may promote and optimize performances of the thrusters due to the simplicity and suitability of the power-law fluid for analyzing a wide range of fluids. In recent years, numerous researchers have investigated the flow of power-law fluids in micro/nano channels [18,19,20,21,22,23]. Zhao et al. [24] studied the electro-seepage velocity of power-law fluids in a slit microchannel and acquired exact solutions of the flow velocity for different flow behavior indices, as well as approximate solutions with the approximate scheme of the hyperbolic sine/cosine function. Choi et al. [25] obtained analytical solutions of the electric potential for the power-law fluid by solving the Poisson–Boltzmann equation under the Debye–Hückel linearization principle so that the velocity expressions of the EOF are calculated in planar microchannels. Moreover, Shit et al. [26] developed the power-law fluid model to study EOF and heat transfer in a microchannel considering the effects of Joule heat and viscous dissipation, which has significance for the non-Newtonian pseudoplastic fluid and dilatant fluid. Based on features of the porous fiber media, the permeability of power-law fluids was explored by Zhu et al. [27] who obtained analytical solutions of the pressure-driven flow under the effect of the EDL.

Previous research that pertained to the micro/nano electro-osmotic thrusters only considered the effect of Newtonian fluids on thruster performance, so our purpose was to investigate the influences of non-Newtonian power-law fluids with different flow behavior indices on thruster performance. A power-law fluid thruster model in a microchannel was considered here for the first time, and the influence of its flow behavior index on the thruster performance was studied theoretically. By solving the Poisson–Boltzmann equation and the modified Navier–Stokes equation, analytical solutions for the electric potential and electro-osmotic velocity were obtained under the hypothesis of the Debye–Hückel linearization and the approximate scheme of the hyperbolic sine/cosine function. The analytical velocity result was valid due to its consistency with the numerical simulation result. Then, specific impulse and thruster thrust generated by the power-law fluid flow were calculated; additionally, thruster efficiency and the thrust-power ratio were given after obtaining the total input powers, which consisted of kinetic energy per unit time, Joule heat, viscous dissipation, and frictional heating on the walls. Moreover, we found a good result indicating that features of the non-Newtonian pseudoplastic fluid are able to promote the development of these performance parameters, which is discussed in detail.

## 2. Electrodynamic Mathematical Model

**Figure 1:**
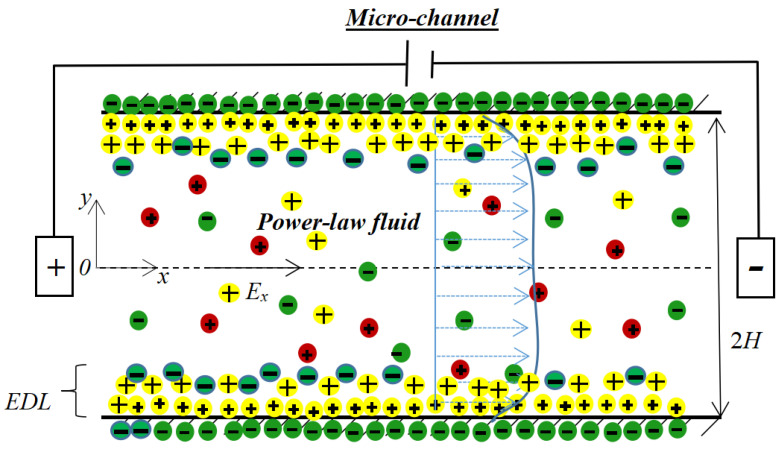
Schematic diagram of microchannel in the micron electric thruster.

In Figure 1 below, we show the geometric and physical arrangement of the microchannel of the power-law fluid electro-osmotic thrusters. The electrolytic propellant in the channel is an incompressible non-Newtonian power-law fluid that is considered to be an ideal solution of a completely separated, symmetrical salt, which is first stored in a liquid storage tank and then extracted from the appropriate inlet into the thruster. An electric field with a field intensity of *E_x_* drives a power-law fluid that electrifies the electrode to generate fluid flow and then thrust, as well as energy. As the applied electric field provides driving forces for the fluid, a part of the input power is consumed by the entire system from the power processing unit to maintain the continuous operation of the thruster. The thruster is regarded as a parallel-plate or slit-micron channel with length, width, and depth of *L*, *W*, and 2*H*, respectively, as shown in Figure 1. We assume that the length and depth of the channel are much greater than the height; that is, *L* and *W* >> *H*, so the flow field is simplified to be single-phase and inertia-free. Electrokinetic effects occur at the interface between the dilute aqueous electrolyte solution and the non-conductive wall surface. Moreover, the temperature change on the channel cross section is negligible compared to the absolute temperature.

### 2.1. Electric Potential and Ionic Concentration Distributions

The electrostatic potential distribution obeys the Poisson equation, and its expression is

(1) $$\frac{d^{2}\psi }{dy^{2}}=-\frac{\rho_{e}}{\varepsilon },\;0\leq y\leq H$$

where *ψ*(*y*) is the electric potential distribution, *ε* = *ε*_0_*ε_r_*, *ε*_0_ represents the electric permittivity of free space, and *ε_r_* stands for the relative permittivity. The volumetric net charge density of the fluid is denoted as *ρ_e_* and is written as

(2) $$\rho_{e}=ez(n^{+}-n^{-}),$$

where *e* is the proton charge, *z* stands for the valence number of ions, and *n*^+^ and *n*^−^ denote the ionic number concentration of cations and anions. If the electrolyte ions follow the Boltzmann distribution, the following equation is used:

(3) $$n^{\pm }=n_{0}\exp(\mp \frac{ez}{k_{b}T_{av}}\psi ),$$

where the bulk number concentration *n*_0_ is ion density, *k_b_* stands for the Boltzmann constant, and *T_av_* denotes the absolute temperature of the system. Substituting Equations (2) and (3) into Equation (1) leads to the Poisson–Boltzmann (P-B) equation, which is as follows:

(4) $$\frac{d^{2}\psi }{dy^{2}}=\frac{2n_{0}ez}{\varepsilon }sinh(\frac{ez}{k_{b}T_{av}}\psi ),\;0\leq y\leq H$$

To linearize the above equation and then obtain an analytical solution, the Debye–Hückel approximation sinh((*ez*/*k_b_T_av_*)*ψ*)~(*ez*/*k_b_T_av_*)*ψ* is employed when |(*ez*/*k_b_T_av_*)*ψ*| <1 or |*ψ*| < 25 mV at the average temperature *T_av_* = 300 *K*. Thus, assuming a low electric potential in the channel, the P-B equation is simplified as follows:

(5) $$\frac{d^{2}\psi }{dy^{2}}=\kappa^{2}\psi ,\;0\leq y\leq H$$

where *κ* = 1/*λ_D_*, κ is the reciprocal of the EDL thickness or the electrokinetic width, *λ_D_* = [*εk_b_T_av_*/(2*n*_0_*e*^2^*z*^2^)]^1/2^, and λ_D_ stands for the EDL thickness. The boundary conditions of the Poisson–Boltzmann (P-B) equation are as follows:

(6) $${\frac{d\psi }{dy}|}_{y=0}=0,$$

(7) $${\psi |}_{y=H}=\zeta ,$$

where *ζ* denotes the electric potential at the wall. The maximum value of the zeta potential *ζ* on the wall is selected as −25 mV to satisfy the D-H linearization principle. Equation (6) satisfies the symmetry of the electrolyte solution, and Equation (7) represents the Gaussian boundary condition of the channel wall. To facilitate the solution, we introduce some dimensionless variables:

(8) $$\psi^{*}=\frac{\psi }{\zeta },\;y^{*}=\frac{y}{H},\;\kappa^{*}=\kappa H,$$

The analytical solution of Equations (5)*–*(7) in dimensionless forms can be expressed as

(9) $$\psi^{*}(y^{*})=\frac{\cosh(\kappa^{*}y^{*})}{\cosh(\kappa^{*})}$$

### 2.2. Fluid Velocity Distribution

In the case of a stable and fully developed unidirectional flow of power-law fluids driven only by an electric field E_x_ and without a pressure gradient or inertia term, the Navier–Stokes (N-S) equation is expressed as

(10) $$\frac{d}{dy}(\mu \frac{du}{dy})+\rho_{e}E_{x}=0,\;0\leq y\leq H$$

As a generalized Newtonian fluid, the viscosity μ of the power-law fluid can be expressed as

(11) $$\mu =m|\frac{du}{dy}{|}^{n-1}=m{\left(-\frac{du}{dy}\right)}^{n-1},$$

where *m* is the flow consistency index, and *n* stands for the flow behavior index. It is noted that different values of the flow behavior index represent two kinds of non-Newtonian power-law fluids, pseudoplastic fluids (*n* < 1) and dilatant fluids (*n* > 1). If the fluid is thought of as a Newtonian fluid, the flow behavior index is equal to one (*n* = 1), according to Equation (11). Next, substituting Equations (1) and (11) into Equation (10), the Navier–Stokes (N-S) equation is rewritten as

(12) $$\frac{d}{dy}[m{\left(-\frac{du}{dy}\right)}^{n-1}\frac{du}{dy}]-\varepsilon E_{x}\frac{d^{2}\psi }{dy^{2}}=0,\;0\leq y\leq H$$

The relevant boundary conditions are as follows:

(13) $${\frac{du}{dy}|}_{y=0}=0,$$

(14) $${u|}_{y=H}=-\beta {\frac{du}{dy}|}_{y=H},$$

in which *β* is slip length. We introduce some dimensionless variables:

(15) $$u^{*}=\frac{u}{u_{HS}},\;\beta^{*}=\frac{\beta }{H},\;u_{HS}=-\frac{\varepsilon E_{x}\zeta }{\mu },$$

where *u_HS_* is the Helmholtz–Smoluchowski or maximum electro-osmotic velocity [28,29]. Hence, Equation (12) is derived in the following form:

(16) $$\frac{du^{*}}{dy^{*}}=-\kappa^{{*}^{\frac{1}{n}}}{\left[\frac{\sinh(\kappa^{*}y^{*})}{cosh(\kappa^{*})}\right]}^{\frac{1}{n}},\;0\leq y^{*}\leq 1$$

When only *n* = 1, 1/2, 1/3, …, Equation (22) is integrated analytically. For other values of *n*, Equation (16) can be approximately treated. We approximate the hyperbolic sine function as follows:

(17) $$\sinh(\kappa^{*}y^{*})=\{\begin{array}{c} \kappa^{*}y^{*},0\leq \kappa^{*}y^{*}\leq 1\\ \frac{1}{2}e^{\kappa^{*}y^{*}},1\leq \kappa^{*}y^{*}\leq \kappa^{*} \end{array},$$

Using the above approximation method, velocity distributions in the electrolyte solution are obtained [24,26]. The dimensionless velocity u* is assumed to be continuous in the interval 0 ≤ *κ* * *y* * ≤ 1 and 1 ≤ *κ* * *y* * ≤ κ* and is expressed as

(18) $$u^{*}=\{\begin{array}{c} u_{1}^{*},0\leq \kappa^{*}y^{*}\leq 1\\ u_{2}^{*},1\leq \kappa^{*}y^{*}\leq \kappa^{*} \end{array},$$

By substituting Equations (18) and (17) into Equation (16), analytical expressions of the non-dimensional velocity are acquired using the boundary conditions (13) and (14) in dimensional forms:

(19) $$u_{1}^{*}(y^{*})=A+B[C-\frac{{\left(\kappa^{*}y^{*}\right)}^{1+\frac{1}{n}}}{n+1}],\;0\leq y^{*}\leq \frac{1}{\kappa^{*}}$$

(20) $$u_{2}^{*}(y^{*})=A+B\frac{e^{\frac{\kappa^{*}}{n}}-e^{\frac{\kappa^{*}}{n}y^{*}}}{2^{\frac{1}{n}}},\;\frac{1}{\kappa^{*}}\leq y^{*}\leq 1$$

with

(21) $$A=\beta^{*}{\left[\frac{\kappa^{*}e^{\kappa^{*}}}{2\cosh(\kappa^{*})}\right]}^{\frac{1}{n}},\;B=\frac{n\kappa^{{*}^{\frac{1}{n}}}}{\kappa^{*}\cosh^{\frac{1}{n}}(\kappa^{*})},\;C=\frac{e^{\frac{\kappa^{*}}{n}}-e^{\frac{1}{n}}}{2^{\frac{1}{n}}}+\frac{1}{n+1}.$$

Noticeably, *u*_1_* and *u*_2_* are equal when y* is equal to 1/κ*.

## 3. Thruster Performance Analysis

Based on the electric potential and velocity of the electro-osmotic micro-thrusters, a number of thruster properties can be derived: specific impulse *I_sp_*, thrust *Th*, efficiency *η*, and thrust-to-power *TP*.

### 3.1. Specific Impulse

Specific impulse is defined as the propellant exhaust velocity divided by the gravitational acceleration constant [30,31]. The propellant exhaust velocity is regarded as the average flow velocity [10,11,12,13]. Thus, the specific impulse, *I_sp_*, is expressed as

(22) $$I_{sp}=\frac{1}{g_{0}H}\int_{0}^{H}u(y)dy=\frac{1}{g_{0}H}[\int_{0}^{\lambda_{D}}u_{1}(y)dy+\int_{\lambda_{D}}^{H}u_{2}(y)dy],$$

or in dimensionless form:

(23) $$I_{sp}^{*}=\int_{0}^{\frac{1}{\kappa^{*}}}u_{1}^{*}(y)dy^{*}+\int_{\frac{1}{\kappa^{*}}}^{1}u_{2}^{*}(y)dy^{*}=I_{sp1}^{*}+I_{sp2}^{*},$$

where *g*_0_ represents the gravitational constant at sea level [30,31] and

(24) $$I_{sp}^{*}=\frac{g_{0}I_{sp}}{u_{HS}},\;I_{sp1}^{*}=\frac{A+BC}{\kappa^{*}}-\frac{Bn}{\left(1+n)(\kappa^{*}+2n\kappa^{*}\right)},\;I_{sp2}^{*}=A+\frac{Be^{\frac{\kappa^{*}}{n}}}{2^{\frac{1}{n}}}-\frac{2^{\frac{1}{n}}A+B[(1+n)e^{\frac{\kappa^{*}}{n}}-ne^{\frac{1}{n}}]}{2^{\frac{1}{n}}\kappa^{*}}.$$

### 3.2. Thrust

During the thruster operation, the transmitted thrust Th is the differential thrust integrated over the cross-sectional area of the channel [15,16,17], as follows:

(25) $$Th=2W\int_{0}^{H}\rho u^{2}(y)dy=2W[\int_{0}^{\lambda_{D}}\rho u_{1}^{2}(y)dy+\int_{\lambda_{D}}^{H}\rho u_{2}^{2}(y)dy],$$

where *ρ* is the fluid density. The thrust can be rewritten in dimensionless form:

(26) $$Th^{*}=\int_{0}^{\frac{1}{\kappa^{*}}}\rho u_{1}^{{*}^{2}}(y^{*})dy^{*}+\int_{\frac{1}{\kappa^{*}}}^{1}\rho u_{2}^{{*}^{2}}(y^{*})dy^{*}=Th_{1}^{*}+Th_{2}^{*},$$

with

(27) $$Th^{*}=\frac{Th}{2WH\rho u_{HS}^{2}},\;Th_{1}^{*}=\frac{1}{\kappa^{*}}\text{\{}A^{2}+2ABC-\frac{2B(A+BC)n}{\left(1+n)(1+2n\right)}+B^{2}[C^{2}+\frac{n}{{\left(1+n\right)}^{2}(2+3n)}]\text{\}}\;Th_{2}^{*}=\frac{2^{-1-\frac{2}{n}}}{\kappa^{*}}\text{\{}2{\left(2^{\frac{1}{n}}A+Be^{\frac{\kappa^{*}}{n}}\right)}^{2}\kappa^{*}-2^{1+\frac{1}{n}}A^{2}-2^{2+\frac{1}{n}}AB[(1+n)e^{\frac{\kappa^{*}}{n}}-ne^{\frac{1}{n}}]-B^{2}[ne^{\frac{2}{n}}-4ne^{\frac{1+\kappa^{*}}{n}}+(2+3n)e^{\frac{2\kappa^{*}}{n}}]\text{\}}$$

### 3.3. Efficiency

Part of the total power input *P_in_* is converted into the power output of the thruster, kinetic energy per unit time *K*, and the rest is dissipated by the Joule heating effect *P_j_*, the viscous dissipation *P_v_*, and the frictional heating *P_f_* [32,33,34,35].

(28) $$P_{in}=K+P_{j}+P_{v}+P_{f},$$

with

(29) $$K=\int_{A_{out}}\frac{1}{2}\rho u^{3}dA_{out}=\int_{A_{out1}}\frac{1}{2}\rho u_{1}^{3}dA_{out}+\int_{A_{out2}}\frac{1}{2}\rho u_{2}^{3}dA_{out},$$

(30) $$P_{j}=\int_{V}\sigma E_{x}^{2}dV,$$

(31) $$P_{v}=\int_{V}\mu {\left(\frac{du}{dy}\right)}^{2}dV=\int_{V_{1}}\mu {\left(\frac{du_{1}}{dy}\right)}^{2}dV+\int_{V_{2}}\mu {\left(\frac{du_{2}}{dy}\right)}^{2}dV,$$

(32) $$P_{f}=\int_{A_{wall}}\mu u_{2}(y=H)\frac{du_{2}(y=H)}{dy}dA_{wall},$$

where

(33) $$\sigma =\sigma_{0}\cosh(\frac{ez}{k_{b}T_{av}}\psi )≅\sigma_{0}[1+\frac{1}{2}{\left(\frac{ez}{k_{b}T_{av}}\psi \right)}^{2}],$$

*σ* stands for the electrical conductivity of the electrolytic propellant, *σ*_0_ represents the electrical conductivity of the neutral liquid, *V* is the total channel volume, *A_out_* is the cross-sectional area of the channel, and *A_wall_* denotes the surface area of the wall.

Their dimensionless forms are given as follows:

(34) $$P_{in}^{*}=K^{*}+P_{v}^{*}+P_{j}^{*}+P_{f}^{*},$$

(35) $$K^{*}=\int_{0}^{\frac{1}{\kappa^{*}}}\frac{1}{2}u_{1}^{{*}^{3}}dy^{*}+\int_{\frac{1}{\kappa^{*}}}^{1}\frac{1}{2}u_{2}^{{*}^{3}}dy^{*}=K_{1}^{*}+K_{2}^{*},$$

(36) $$P_{v}^{*}=\delta^{*}[\int_{0}^{\frac{1}{\kappa^{*}}}{\left(\frac{du_{1}^{*}}{dy^{*}}\right)}^{2}dy^{*}+\int_{\frac{1}{\kappa^{*}}}^{1}{\left(\frac{du_{2}^{*}}{dy^{*}}\right)}^{2}dy^{*}]=\delta^{*}(\Omega_{1}^{*}+\Omega_{2}^{*}),$$

(37) $$P_{j}^{*}=\lambda^{*}[\int_{0}^{1}(1+\frac{1}{2}\alpha^{2}\psi^{{*}^{2}})dy^{*}]=\lambda^{*}j^{*},$$

(38) $$P_{f}^{*}=\delta^{*}[u_{2}^{*}(y^{*}=1)\frac{du_{2}^{*}(y^{*}=1)}{dy^{*}}]=\delta^{*}f^{*},$$

with

(39) $$[K^{*},P_{v}^{*},P_{j}^{*},P_{f}^{*}]=\frac{\left[K,P_{v},P_{j},P_{f}\right]}{2\rho HWu_{HS}^{3}},\lambda^{*}=\frac{L\sigma_{0}E_{x}}{\rho_{e}u_{HS}^{3}},\delta^{*}=\frac{Lmu_{HS}^{n-2}}{\rho_{e}H^{n+1}},\;K_{1}^{*}=\frac{1}{2\kappa^{*}}[A^{3}+3A^{2}BC+3AB^{2}C^{2}+B^{3}C^{3}-\frac{3B{\left(A+BC\right)}^{2}n}{\left(1+n)(1+2n\right)}+\frac{3B^{2}(A+BC)n}{{\left(1+n\right)}^{2}(2+3n)}-\frac{B^{3}n}{{\left(1+n\right)}^{3}(3+4n)}],\;K_{2}^{*}=\frac{2^{-2-\frac{3}{n}}}{3\kappa^{*}}\{-3\times 2^{1+\frac{3}{n}}A^{3}-9\times 2^{1+\frac{2}{n}}A^{2}B[(1+n)e^{\frac{\kappa^{*}}{n}}-ne^{{}^{\frac{1}{n}}}]-9\times 2^{\frac{1}{n}}AB^{2}[ne^{\frac{2}{n}}-4ne^{\frac{1+\kappa^{*}}{n}}+(2+3n)e^{\frac{2\kappa^{*}}{n}}]\;-B^{3}[(6+11n)e^{\frac{3\kappa^{*}}{n}}-18ne^{\frac{1+2\kappa^{*}}{n}}+9ne^{\frac{2+\kappa^{*}}{n}}-2ne^{\frac{3}{n}}]+6\kappa^{*}{\left(2^{\frac{1}{n}}A+Be^{\frac{\kappa^{*}}{n}}\right)}^{3}\},\;\Omega_{1}^{*}=\frac{n\kappa^{{*}^{\frac{1}{n}}}}{\left(1+2n)\cosh^{1+\frac{1}{n}}(\kappa^{*}\right)},\;\Omega_{2}^{*}=\frac{n\kappa^{{*}^{\frac{1}{n}}}\sec h(\kappa^{*})\{e^{\kappa^{*}}{\left[1+\tanh(\kappa^{*})\right]}^{\frac{1}{n}}-e^{1+\frac{1}{n}}\sec h^{\frac{1}{n}}(\kappa^{*})\}}{2^{1+\frac{1}{n}}(1+n)}\;j^{*}=1+\frac{\alpha^{2}}{8}\sec h^{2}(\kappa^{*})[2+\frac{\sinh(2\kappa^{*})}{\kappa^{*}}],\;f^{*}=-\frac{\kappa^{*}e^{\kappa^{*}}A}{2\cosh(\kappa^{*})},$$

Thruster efficiency *η* is the power output, kinetic energy per unit time, divided by the total power input [30,31], written as

(40) $$\eta =\frac{K}{P_{in}}=\frac{K^{*}}{P_{in}^{*}}=\frac{K_{1}^{*}+K_{2}^{*}}{K_{1}^{*}+K_{2}^{*}+\lambda^{*}j^{*}+\delta^{*}(\Omega_{1}^{*}+\Omega_{2}^{*}+f)}.$$

### 3.4. Thrust-to-Power Ratio

The thrust-to-power ratio TP is expressed as

(41) $$TP=\frac{Th}{P_{in}}$$

or in a dimensionless form as

(42) $$TP^{*}=\frac{Th^{*}}{P_{in}^{*}}=\frac{Th_{1}^{*}+Th_{2}^{*}}{K_{1}^{*}+K_{2}^{*}+\lambda^{*}j^{*}+\delta^{*}(\Omega_{1}^{*}+\Omega_{2}^{*}+f)}$$

with

$$TP^{*}=u_{HS}TP.$$

The thrust-to-power ratio is related to the efficiency of the propeller as follows [31]:

(43) $$TP=\frac{Th}{P_{in}}∼\frac{\rho_{e}HWv_{m}^{2}}{\rho_{e}HWv_{m}^{3}/2}=\frac{2\eta }{v_{m}}$$

in which *v_m_* is the average velocity.

## 4. Results and Discussion

In this section, we evaluate the propulsion performance of electro-osmotic thrusters by identifying the effects of relevant parameters on the specific impulse *I_sp_* (ms), the thrust *Th* (μN), the efficiency *η* (%), and the thrust-to-power ratio *TP* (mN W^−1^). Thruster dimensions, characteristics, and fixed parameters are shown in Table 1. The axial electric field *E_x_* was set as 2.2 × 10^6^ V m^−1^ [14,15,16,17,31]. The maximum value of the zeta potential on the walls *ζ* was selected as −25 mV in the study to satisfy the D-H linearization hypothesis. The electrical conductivity *σ*_0_ of the power-law fluid was selected as 10^−3^ S m^−1^ at the average absolute temperature *T_av_* = 300 K. We considered the effects of some parameters on thruster performance in the microchannel, such as the flow behavior index *n*, the reciprocal of the EDL thickness *κ*, and the slip length *β*, practical ranges of which are shown in Table 2.

**Figure 2:**
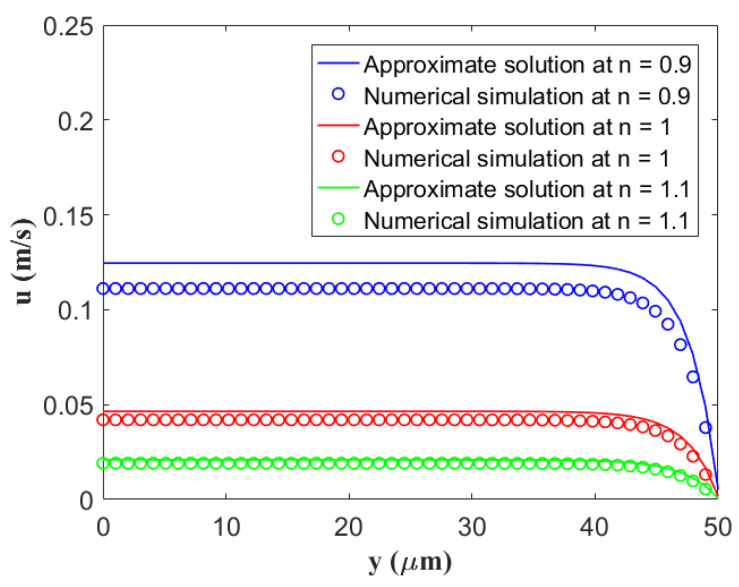
Comparison between the current approximate analytical solutions and numerical simulation solutions for the velocity distribution in the microchannel with regard to different behavior indices *n* by setting fixed values of *κ* = 0.4 μm^−1^, *β* = 100 nm, *ζ* = −25 mV, and *z* = 1.

To verify the validity of the present analytical solutions for flow velocity in the electro-osmosis micro-thruster, we first compared them with the numerical simulation results subjected to different behavior indices *n* in Figure 2. The numerical simulation solutions for fluid velocity were obtained by the finite difference method. Figure 2 shows that the current velocity distributions have a good agreement with the numerical solutions.

**Figure 3:**
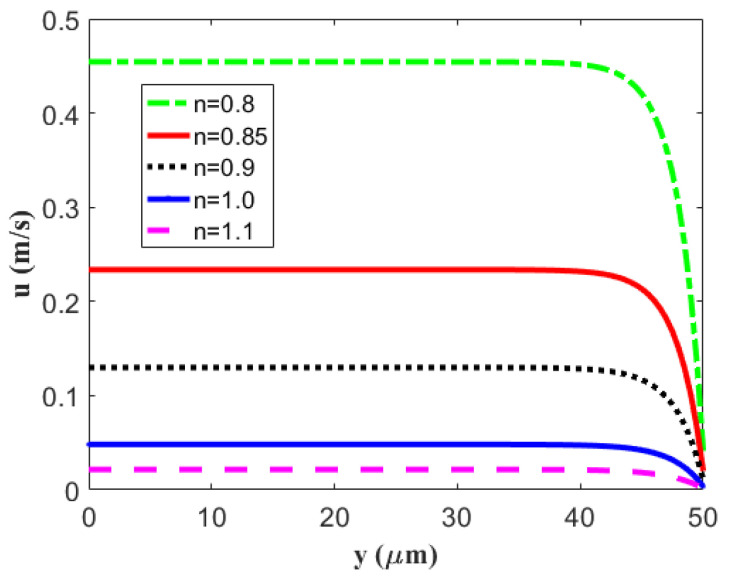
The velocity distribution of the electro-osmosis thruster in the microchannel with regard to a different flow behavior index *n* by setting fixed values of *κ* = 0.4 μm^−1^, *β* = 200 nm, *ζ* = −25 mV, and *z* = 1.

In Figure 3, we demonstrate the velocity distribution of a power-law fluid at the half height of a rigid micron channel for different flow behavior exponents *n*. To intentionally highlight the difference between flow velocities of pseudoplastic fluids (*n* < 1) and dilatant fluids (*n* > 1), the velocity profile is presented at a space [26]. Under the condition of fixing other parameters, the velocity of the pseudoplastic fluid is greater than the velocity of the Newtonian fluids (*n* = 1), while the velocity of the dilatant fluid is smaller than that of the Newtonian fluid. That is to say, the velocity increases with the decrease in the flow characteristic index.

**Figure 4:**
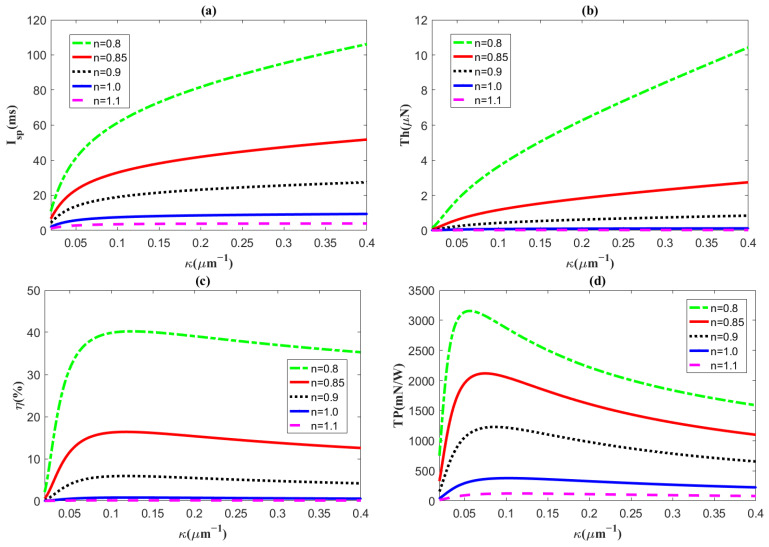
The distributions of (**a**) specific impulse *I_sp_*, (**b**) thrust *Th*, (**c**) efficiency *η*, and (**d**) thrust-to-power ratio *TP* plotted, corresponding to the reciprocal of the EDL thickness *κ* for different flow behavior indices *n*, with the slip length *β* = 200 nm, *ζ* = −25 mV, and *z* = 1.

Figure 4 evaluates the variations in the specific impulse *I_sp_*, thrust *Th*, thruster efficiency *η,* and thrust-to-power ratio *TP* with the flow behavior index *n*, using the reciprocal of the EDL thickness *κ* as the abscissa. It is expected that, in Figure 4a–c, the specific impulse *I_sp_* and thrust *Th* increase with the electrokinetic width *κ*, and that the thruster efficiency *η* first increases and then flattens gradually with it for a fixed flow behavior index. The cause is that the larger the electrokinetic width, the larger the ion number concentration in the solution, leading to a higher flow velocity. The enhancement in the electro-osmotic velocity promotes development of the specific impulse, thrust, and thruster efficiency. When the electrokinetic width is constant, the three of them decrease with the flow behavior index. The above results are consistent with the results shown in Figure 3, which demonstrate that the velocity decreases with the flow behavior index.

We can further explain the results in Figure 4a–c, which show that the specific impulse and thrust are proportional to the velocity and the square of the velocity, respectively, according to Equations (22) and (25). That is to say, as the flow velocity is enhanced, the specific impulse and thrust are improved, too. Moreover, in Equation (29), kinetic energy of the electrolytic propellant *K* is also proportional to the cube of the velocity. Hence, with a gradual increase in the thruster velocity, the growth rate of the kinetic energy is greater than the growth rate of the dissipated items generated by the Joule heating effect *P_j_*, viscous dissipation *P_v_*, and frictional heating *P_f_*, which leads to thruster efficiency growth.

Particularly, in the last part of Figure 4, the thrust-to-power ratio increases first and then decreases with the electrokinetic width for the flow behavior index *n* < 1. When the electrokinetic width increases, the efficiency in Equation (43) as a numerator increases rapidly, but the denominator, the flow velocity, eventually grows faster than the numerator, resulting in a trend of thrust-to-power ratio. It is obvious that, in micro electro-osmotic thrusters, the pseudoplastic fluid (*n* < 1) performs better than Newtonian fluids (*n* = 1) and dilatant fluids (*n* > 1) do, and the dilatant fluid is too weak to be used as a thruster solvent. Thus, the pseudoplastic fluid is most suitable as a propeller solvent among the three kinds of fluids, since its properties are higher than those of the Newtonian fluid and the dilatant fluid.

**Figure 5:**
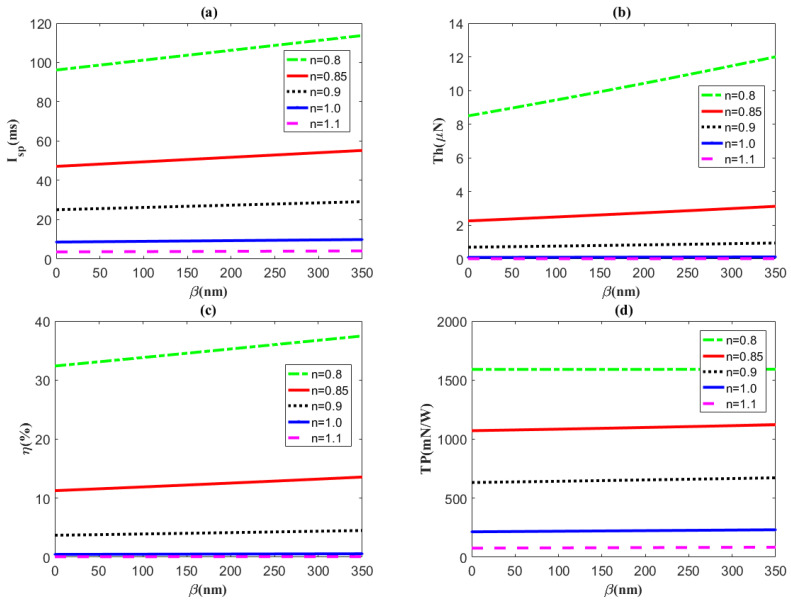
The distributions of (**a**) specific impulse *I_sp_*, (**b**) thrust *Th*, (**c**) efficiency *η*, and (**d**) thrust-to-power ratio *TP* plotted against slip length *β* for different flow behavior indices *n* with the electrokinetic width *κ* = 0.4 μm^−1^, *ζ* = −25 mV, and *z* = 1.

Figure 5 presents the variations in these performances with regard to the slip length *β* for different flow behavior indices. Evidently, most of the changes with changing slip length are not noticeable since nanoscale slippage has few influences on fluid motion in a microchannel. However, for the pseudoplastic fluid (*n* < 1, especially *n* = 0.8), the specific impulse, thrust, and thruster efficiency significantly increase with the slip length. This is a noteworthy result because we can enhance thruster thrust and efficiency by increasing the slip length for the pseudoplastic fluid.

## 5. Conclusions

In this article, the power-law fluid thruster in a microchannel was first considered, and the influence of the flow behavior index of the power-law fluid on thruster performance was studied theoretically. By solving the Poisson–Boltzmann equation and the modified Navier–Stokes equation, the analytical solutions for the potential and velocity were obtained under the hypothesis of the Debye–Hückel linearization and the approximate scheme of hyperbolic sine function. The present analytical solution for flow velocity is valid because it has a good agreement with the numerical solution. Then, the specific impulse, thruster thrust, and total input power, which are generated by the electro-osmotic velocity, were calculated so that thruster efficiency and thruster power ratio were given. It was found that the specific impulse, thrust, and thruster efficiency increase with the reciprocal of the EDL, while the thrust-to-power ratio increases first and then decreases. Moreover, these performance parameters improved significantly when the non-Newtonian power-law fluid was the pseudoplastic fluid. That is to say, in micro electro-osmotic thrusters, the non-Newtonian pseudoplastic fluid performed better than Newtonian fluids and dilatant fluids did; thus, it is most suitable as a propeller solvent. The specific impulse, thrust, and thruster efficiency significantly increased with the slip length of the nanoscale for the pseudoplastic fluid (especially *n* = 0.8) in the microchannel.

## Figures and Tables

**Table 1:** Numerical values of the thruster dimensions, properties, and fixed parameters.

Symbol	Description	Value	Units	References
*L*	thruster length	1	mm	[15,29,30,31]
*W*	thruster depth	500	μm	[15,29,30,31]
*H*	half channel height of the thruster	50	μm	[15,29,30,31]
*ρ*	density of the fluid	1060	kg m^−3^	[26]
*ε*_0_	permittivity of free space	8.854 × 10^−12^	F m^−1^	[16]
*ε_r_*	relative permittivity	78.36	F m^−1^	[16]
*σ*_0_	electrical conductivity of the fluid	10^−3^	S m^−1^	[26]
*z*	valence number of ions	1		[26]

**Table 2:** Practical number ranges of the main operating parameters and EDL specifications.

Parameter	Description	Value	References
*n*	flow behavior index	0.8–1.2	[23,25,26]
*κ*	reciprocal of EDL thickness	0–0.5 μm^−1^	[23,24,25]
*β*	slip length	0–350 nm	[16,36]

## Data Availability

Data sharing not applicable.

## Nomenclature

*L*	length of the channel, mm
*W*	width of the channel, μm
*H*	depth of the channel, μm
*E_x_*	Electric field strength, V m^−1^
*x*, *y*	two-dimensional coordinate components, μm, μm, respectively
*x* *, *y* *	dimensionless two-dimensional coordinate components
*e*	proton charge = 1.602 × 10^−19^ °C
*z*	valence number of ions
*n*_0_	ion density, m^−3^
*K_b_*	Boltzmann constant = 1.38 × 10^−23^ J K^−1^
*T_av_*	absolute temperature of the system, K
*u*	fluid velocity, m/s
*u**	dimensionless fluid velocity
*u_HS_*	Helmholtz–Smoluchowski electro-osmotic velocity, m/s
*m*	flow consistency index
*n*	flow behavior index
*I_sp_*	specific impulse of thruster, s
*I_sp_**	dimensionless specific impulse of thruster
*Th*	thrust of thruster, N
*Th**	dimensionless thrust of thruster
*P_in_*	total power input, W
*P_in_**	dimensionless total power input
*K*	kinetic energy per unit time, W
*K**	dimensionless kinetic energy per unit time
*P_j_*	Joule heating effect, W
*P_j_**	dimensionless Joule heating effect
*P_v_*	viscous dissipation, W
*P_v_**	dimensionless viscous dissipation
*P_f_*	frictional heating, W
*P_f_**	dimensionless frictional heating
*TP*	thrust-to-power of thruster, N W^−1^
*TP**	dimensionless thrust-to-power of thruster
*Greek symbols*	
*ψ*	electric potential of fluid, V
*ψ**	dimensionless electric potential of fluid
*ε*_0_	electric permittivity of free space, F m^−1^
*ε_r_*	relative permittivity, F m^−1^
*ρ_e_*	volumetric net charge density of the fluid, cm^−3^
*λ_D_*	EDL thickness, μm
*κ*	reciprocal of the EDL thickness, μm^−1^
*κ**	dimensionless reciprocal of the EDL thickness
*ζ*	electric potential at the wall, *V*
*μ*	viscosity of the power-law fluid, kg/(ms)
*β*	slip length, nm
*ρ*	fluid density, kg m^−3^
*σ*	electrical conductivity of electrolytic propellant, S m^−1^
*σ*_0_	electrical conductivity of the neutral liquid, S m^−1^
*η*	thruster efficiency, %
